# Virus-like particles containing multiple antigenic proteins of *Toxoplasma gondii* induce memory T cell and B cell responses

**DOI:** 10.1371/journal.pone.0220865

**Published:** 2019-08-29

**Authors:** Su-Hwa Lee, Ki-Back Chu, Hae-Ji Kang, Fu-Shi Quan

**Affiliations:** 1 Department of Biomedical Science, Graduate School, Kyung Hee University, Seoul, Republic of Korea; 2 Department of Medical Zoology, Kyung Hee University School of Medicine, Seoul, Republic of Korea; 3 Department of Medical Research Center for Bioreaction to Reactive Oxygen Species and Biomedical Science Institute, School of Medicine, Graduate school, Kyung Hee University, Seoul, Republic of Korea; Instituto Butantan, BRAZIL

## Abstract

Although the efforts to develop vaccine against *Toxoplasma gondii* infection were made for decades, there is currently no licensed vaccine available for humans. Upon discovering a number of T or B cell epitope regions from *T*. *gondii* IMC, ROP18 and MIC8, multi-antigen VLPs or combination VLPs were generated. Mice immunized with multi-antigen VLPs or combination VLPs were challenge infected with *T*. *gondii* (ME49). *T*. *gondii*-specific IgG, IgG isotypes and IgA antibody responses, memory T and B cell responses and protection were evaluated. All the mice survived upon *T*. *gondii* challenge infection by multi-antigen VLPs vaccination. Vaccinated mice elicited higher levels of parasite-specific IgG and IgG2a antibody responses in sera, IgA antibody responses in feces, CD4^+^ and CD8^+^ T cell responses, and cytokines (IFN-γ, IL-10) responses compared to combination VLPs. In particular, the multi-antigen VLPs vaccination showed significantly higher levels of antibody secreting cell (ASC) responses, CD4^+^ and CD8^+^ effector memory T cells, and memory B cells than combination VLPs. Multi-antigen VLPs vaccination showed significant reduction of brain cyst counts and size, and all mice survived. Prediction and analysis of epitopes have indicated that IMC, ROP18 and MIC8 showed partially overlapping epitopes for T and B cells. Our results indicated that antibody responses, memory T and B cells induced by multi-antigen VLPs vaccination might contribute to the complete protection upon *T*. *gondii* (ME49) challenge infection.

## Introduction

*Toxoplasma gondii*, a notorious protozoan parasite belonging to the phylum *Apicomplexa*, is capable of infecting virtually all vertebrate hosts using various transmission routes to cause the zoonotic disease toxoplasmosis [1,2]. Although large variations in the prevalence rates of human toxoplasmosis do exist between countries, it is presumed that approximately third of the world’s population have been infected by *T*. *gondii* [1,3]. Clinical symptoms associated with the disease in immunocompetent individuals are often asymptomatic or of non-specific origin, which includes myalgia, fever, and other flu-like symptoms [1,2]. However, *T*. *gondii* infection can have severe health consequences in pregnant individuals, as these parasites can traverse through the placenta to cause premature abortion and other congenital defects [2,3]. Therapeutic regimen for human toxoplasmosis requires the use of pyrimethamine and sulfadiazine, but side effects and insufficient efficacies against non-tachyzoite stages of the parasite limits their use [4]. Toxovax is currently the only available commercial toxoplasmosis vaccine, albeit being limited to veterinary use with arising safety concerns [5]. These issues, combined with other detriments associated with the treatment, may have created an impetus for the development of a novel vaccine which could effectively block and control the transmission of toxoplasmosis.

The importance of bioinformatics and its growing usage for epitope predictions and vaccine design strategies cannot be overstated. Several *T*. *gondii* vaccine studies have already performed *in silico* epitope analyses of multiple candidate antigens, which may substantially contribute to future vaccine design strategies [6–9].

Vaccination induced immunological memory responses are critically important in inducing protection against the same pathogen recognized by immune system [10,11]. Naïve CD4^+^ T cells recognize antigen-MHC complexes and proliferate and differentiate to effector T cells, which provide immediate protection [12]. Although most of the effector T cells subsequently die by apoptosis, a subset of antigen-specific T cells will persist in immune system as memory T cells once pathogens have been eliminated from the host [13]. Multiple memory T cell subpopulations, including but not limited to central memory T cells (T_CM_) and effector memory T cells (T_EM_), have been identified in humans as of current which can be distinguished based on CD45RO and CD45RA isoform expressions [12]. The T_CM_ shows self-renewal potential and resides in secondary lymphoid organs but lacks effector function, whereas T_EM_ possess immediate effector functions and can rapidly immigrate to peripheral tissues to provide antigen elimination [14]. Increased central memory lymphocyte response induction was observed in cattle vaccinated against the parasite *Theileria parva* using Tp1 antigen post-challenge [15]. Memory B cells (MB) and plasma cells are the key for maintaining humoral immune response [16]. Microneedle delivery of influenza vaccines have been reported to induce a durable, antigen-specific MB and plasma cell responses in mice [17,18].

Recombinant protein and DNA vaccine studies using potential candidate antigens have been conducted extensively in the past [19–21]. Yet, the vaccine efficacies in the aforementioned studies were extremely limited and complete protection was not conferred in mice [22]. Our previous works using virus-like particle vaccines containing single IMC, ROP18, MIC8, ROP4 proteins or multiple proteins have conferred complete protection against *T*. *gondii* [22–26]. These studies mainly focused on inducing *T*. *gondii*–specific IgG, IgA antibodies, CD4^+^ T cells, CD8^+^ T cell responses, and protections in mice [22–26]. Exemplified by earlier works, multi-antigen containing VLPs showed higher number of VLPs particles per μm^2^, higher levels of CD4^+^ T cell, and germinal center B cell responses, which resulted in better protection [25]. To date, immunological memory T or B cell responses induced by single or multiple proteins containing VLPs, T or B cell epitopes in VLPs and antibody secreting cell response induced upon *T*. *gondii* ME49 challenge infection has yet to been reported. As such, in this study, we report the memory T and B responses, T or B cell epitopes, antibody secreting cell (ASC) responses and protections induced by multi-antigen VLPs and combination VLPs upon *T*. *gondii* ME49 challenge infections in mice.

## Materials and methods

### Ethics approval and consent to participate

All experimental procedures involving animals were reviewed and approved by Kyung Hee University IACUC (permit number: KHUASP (SE) - 18–050). Animals were housed in pathogen-free animal facility with easy access to food and water. All of the researchers involved in the study were trained for proper animal handling.

### Parasite preparation, cells, and antibody acquisition

Parasites were maintained in mice as previously described [8,27–29]. Briefly, female BALB/c mice purchased from KOATECH (Pyeongtaek, South Korea) were used, where RH and ME49 were maintained via serial intraperitoneal and oral passage, respectively [29]. Recombinant baculovirus (rBV) and virus-like particles (VLPs) were produced using *Spodoptera frugiperda* Sf9 cells, which were cultured in spinner flasks at 27°C, 130–140 rpm with SF900 II medium (Invitrogen, Carlsbad, CA, USA). Polyclonal *T*. *gondii* antibodies were acquired from sera of *T*. *gondii* (ME49)-infected mice. Horseradish peroxidase (HRP)-conjugated secondary antibodies and monoclonal mouse anti-M1 antibody wae purchased from Southern Biotech (Birmingham, AL, USA) and Abcam (Cambridge, UK), respectively.

### Generation and characterization of VLPs

Inner membrane complex (IMC; accession number: HQ012579, 495bp), rhoptry protein 18 (ROP18; accession number: AM075204, 1,665bp), microneme protein 8 (MIC8; accession number: AF353165, 2,055bp) of *T*. *gondii*, and influenza matrix protein 1 (M1; accession number: EF467824, 1,027bp) genes were cloned into pFastBac vectors and sequenced as described previously [22,26,27]. Following further transformation into DH10Bac competent cells, recombinant baculoviruses expressing the 4 genes were produced as previously described [22,25–27]. Combination VLPs expressing the three proteins (IMC, MIC8, and ROP18) were generated using VLP expressing each of the protein along with influenza M1 as core protein, as described previously [22,26,27]. Multi-antigen VLPs were constructed by co-infecting Sf9 cells with the rBVs together with influenza M1. Sf9 cell culture supernatants were harvested 3 days post—infection, and cellular debris were removed by centrifugation at 6,000 rpm for 40 min at 4°C. VLPs were purified by sucrose gradient as described previously and stored at 4°C until use [22,26,27]. VLPs concentration was determined using QuantiPro BCA assay Kit (Sigma-Aldrich, St, Louis, MO, USA). VLPs were subsequently characterized using transmission electron microscope (TEM) and western blot, and the acquired data were identical to those described in our previous study [25].

### Prediction of T cell and B cell epitopes

Amino acid sequence of *T*. *gondii* IMC (accession number: ADV15617), ROP18 (accession number: CAJ27113), and MIC8 (accession number: AAK19757) were retrieved from NCBI GenBank sequence database. Prediction of T cell and B cell epitopes of IMC, ROP18 and MIC8 was performed using IEDB web server (http://tools.iedb.org). MHC binding affinity, rescale binding affinity, C-terminal cleavage affinity, and transport associated with antigen processing (TAP) transport efficiency propensity scores were calculated for T cell epitope prediction. B cell epitopes were predicted based on the propensity scale method, which evaluates biophysical parameters of the amino acids such as linear epitope, beta-turns, surface exposure, flexibility, antigenicity, and hydrophilicity.

### Immunization and challenge infection in mice

Seven weeks old BALB/c mice were divided into groups (n = 20 per group) for immunization with either multi-antigen VLPs or combination VLPs. On week 0 and 4, mice were intranasally (IN) immunized with 90 μg of multi-antigen VLPs whereas three VLPs expressing IMC, ROP18, or MIC8 were combined at equal ratio (30 μg from each VLP, total 90 μg protein) for combination VLP immunization. Mice were challenge-infected with 500 bradyzoites of ME49 strains via oral route 4 weeks after receiving second immunization. Ten mice were sacrificed from each group at week 4 post-challenge for brain and spleen collection as previously described [22] and brain tissues were prepared for brain cyst counting as previously described [23]. Remaining mice were monitored daily to observe changes in body weight and survival until death. A total of 70 mice were used in the present study, of which 55 were sedated and humanely euthanized by CO_2_ inhalation upon loss of 20% or more body weight post-infection. The entire experiment lasted 3 months, which involved 2 months of immunization phase followed by 1 month of challenge infection period. All efforts have been made to minimize animal suffering and distress, in compliance with the animal welfare policy.

### *T*. *gondii*-specific antibody responses in sera and feces

Sera and feces of mice were collected from all groups 4 weeks post-challenge infection. Fecal antibodies were collected by immersing feces with 500 μl 0.1 M PBS and incubating it at 37°C for 1h, which were subsequently centrifuged at 2,000 rpm for 10 min. Harvested supernatants were stored at -20°C until use. *T*. *gondii*-specific IgG, IgG1, IgG2a and IgA antibody responses were determined by enzyme-linked immunosorbent assay (ELISA) as described previously [22]. Briefly, 96-well flat-bottom immunoplates were coated with 4 μg/mL *T*. *gondii* antigen in 100 μL of 0.05 M carbonate bicarbonate buffer (pH 9.6) and incubated overnight at 4°C. The next day, each wells were incubated with 100 μL of serum samples (diluted 1:100 in PBST) for 2 h at 37°C. HRP-conjugated IgG, IgG1, IgG2a, and IgA secondary antibodies were diluted 1:2000 in PBST and 100 μL of these were added to each well to determine *T*. *gondii*-specific antibody responses.

### Splenocytes preparation and flow cytometry labeling

Spleens were aseptically isolated from ten mice from each group 4 weeks after challenge. Splenocyte suspensions were prepared in RPMI-1640 culture media supplemented with 10% FBS, after RBC lysis with red blood cell lysing buffer hybrid-max (Sigma-Aldrich, St. Louis, MO, USA), for flow cytometry analysis, antibody secreting cell (ASC) assays and cytokine analysis. Cells were stained with trypan blue (Welgene, Daegu, South Korea) and counted with a hemocytometer chamber under a microscope. Splenocytes from each animal were resuspended in staining buffer (2% bovine serum albumin and 0.1% sodium azide in 0.1 M PBS). Cells from individual mouse were separately incubated with Fc Block (clone 2.4G2; BD Biosciences, CA, USA) to block non-specific binding at 4°C for 15 min, and then stained at 4°C for 30 min with different combinations of FITC, PE, PE-Cy5, PE-Cy7 or APC conjugated anti-CD3e (145-2c11), anti-CD4 (GK1.5), anti-CD8a (53–6.7) (BD Biosciences, CA, USA). For memory T cell responses, anti-CD44 (IM7) and anti-CD62L (MEL-14) were used (BD Biosciences, CA, USA) as indicated [28]. For memory B cell responses, anti-CD45R/B220 (RA3-6B2), anti-CD27 (LG-3A10) and anti-IgG1 (A85-1) were used (BD Biosciences, CA, USA) [29]. Events were acquired on BD Accuri C6 Flow Cytometer (BD Biosciences, CA, USA) and data were analyzed using C6 Analysis software (BD Biosciences, CA, USA).

### Antibody secreting cell (ASC) and cytokine analysis

For ASC assays, 96-well culture plates were coated with *T*. *gondii* antigen at 4°C overnight, and splenocytes (10^6^ cells/well) in RPMI-1640 culture media were added to coated plates after blocking. Secreted antibody levels were determined 3 days after incubation at 37°C. To detect cytokines, splenocytes (10^6^ cells/well) were cultured in 96-well culture plates with *T*. *gondii* antigen (5 μg/mL) or medium alone which was used as control at 37°C with 5% CO_2_ for 3 days. The culture supernatants were collected at 72 h for determination of IFN-γ and IL-10. The levels of IFN-γ and IL-10 were examined using BD OptEIA Set according to the manufacturer’s instructions (BD Biosciences).

### Statistics

Statistical analyses were performed using PC-SAS 9.3 software (SAS Institute, Cary, NC, USA). Data sets were expressed as mean +/- SEM and compared using One-way ANOVA with Duncan's *post hoc* analysis. A P value < 0.01 was considered statistically significant.

## Results

### Generation of virus—Like particles

**Fig 1:**
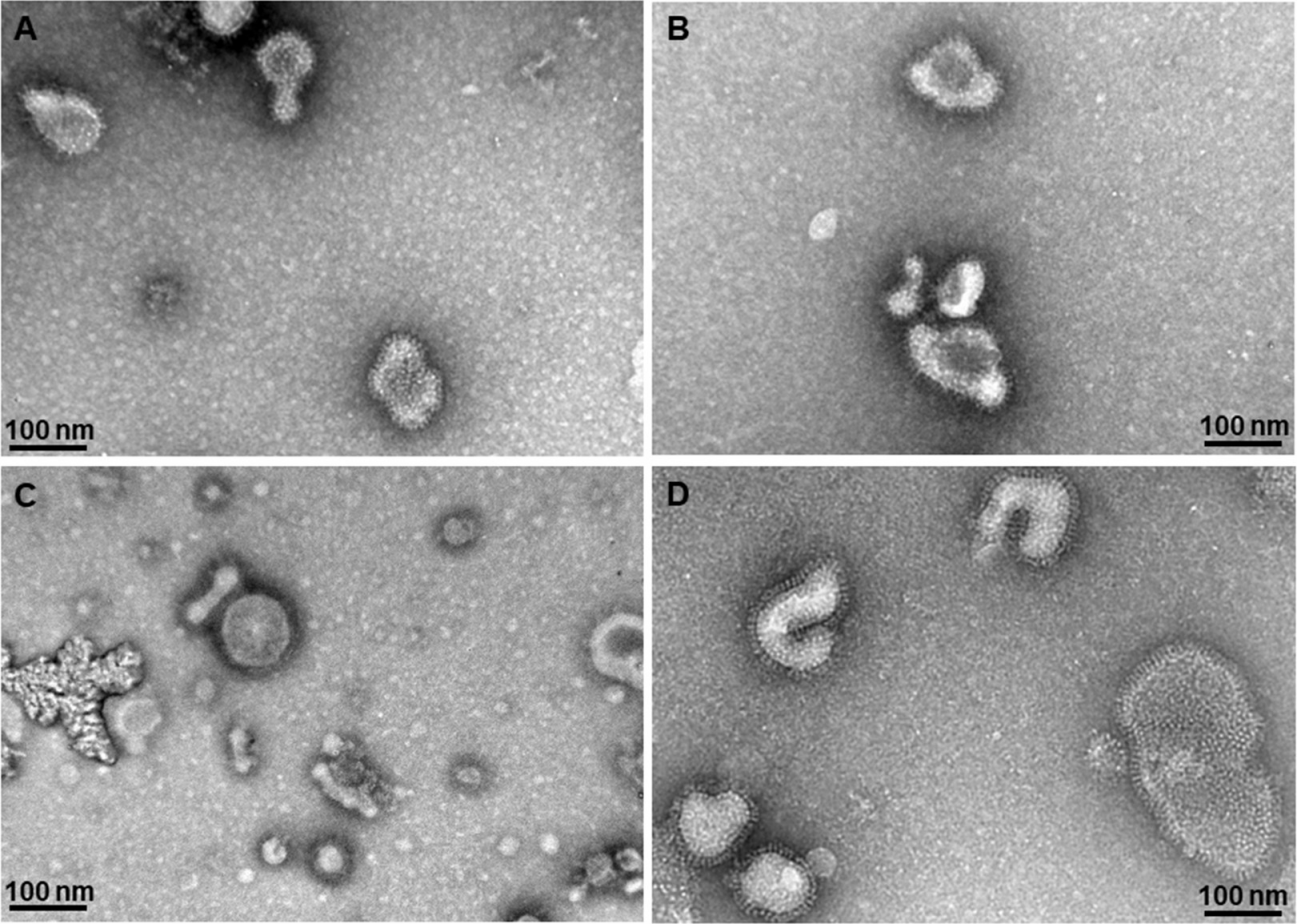
Electron microscopy. *T*. *gondii* IMC VLPs (A), ROP18 VLPs (B), MIC8 VLPs (C), and multi-antigen VLPs (D) were examined under transmission electron microscopy. Spherical morphology of VLPs and exhibited antigen spikes on their surfaces were observed.

VLPs expressing IMC (A), ROP18 (B), MIC8 (C) or multi-antigen VLPs (D) together with influenza M1 were produced as described previously [25] (Fig 1). IMC, ROP18 and MIC8 present 17, 61 and 75kDa of molecular weight [25].

### VLPs vaccination induces *T*. *gondii*-specific IgA, IgG, IgG1 and IgG2a antibody responses

**Fig 2:**
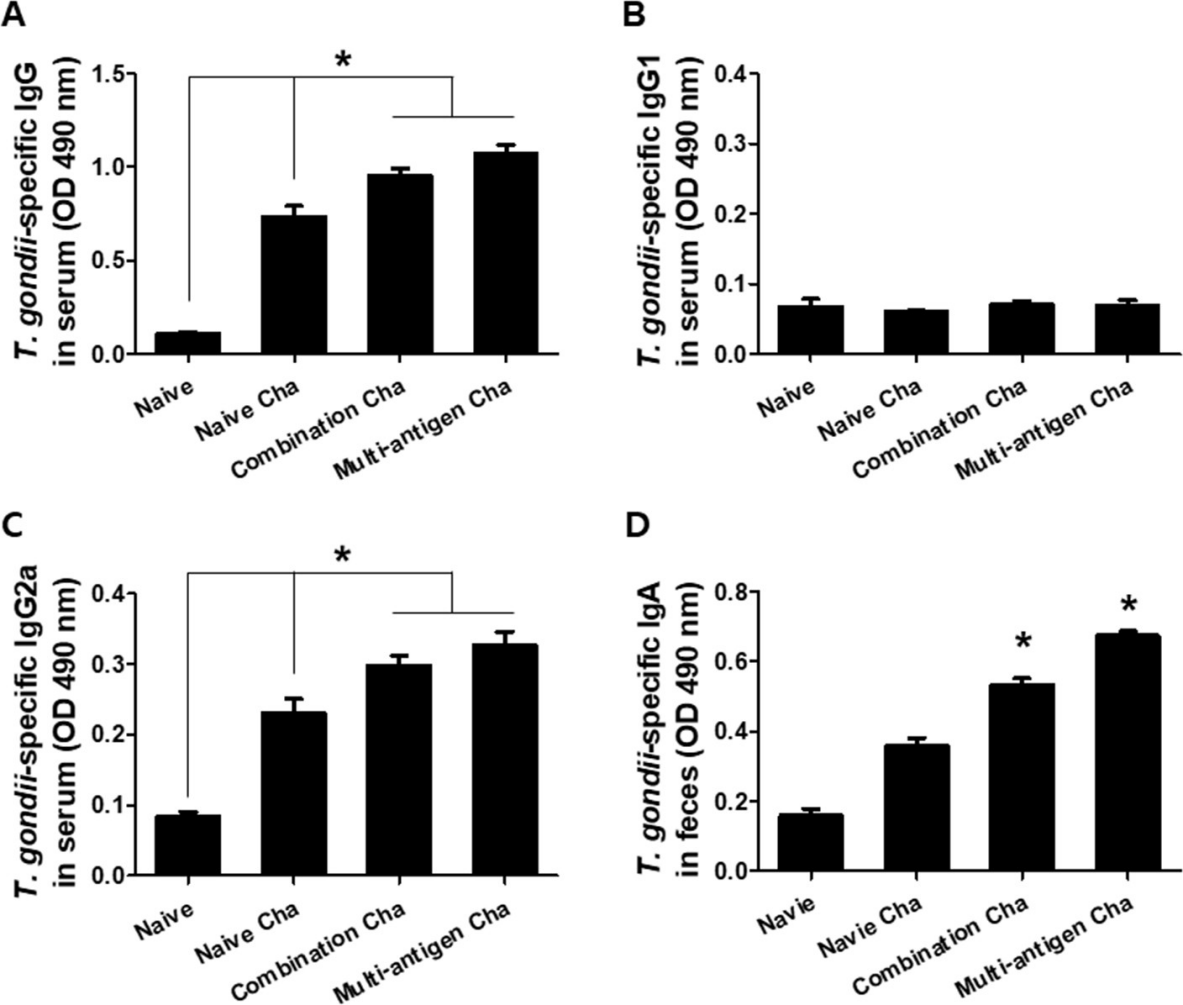
*T*. *gondii*-specific antibody responses. *T*. *gondii*-specific IgG, IgG1 and IgG2a were determined in mice sera after challenge infection. Higher levels of IgG and IgG2a were found in multi-antigen VLPs (Multi-antigen Cha) or combination VLPs (Combination Cha) immunized mice compared to naïve challenged mice (Naïve Cha) (A and C, *P < 0.01). *T*. *gondii*-specific IgA antibody response was determined from mice feces (D). VLPs vaccination showed higher level of IgA response in both Multi-antigen Cha and Combination Cha compared to Naïve Cha (D, *P < 0.01).

To evaluate the level of *T*. *gondii*-specific antibody induced by multi-antigen VLPs or combination VLPs vaccines, we measured the IgG, IgG1 and IgG2a antibody responses in serum and measured the IgA antibody responses in feces upon challenge (Fig 2). Multi-antigen VLPs or combination VLPs immunized group (Multi-antigen Cha or Combination Cha) showed higher levels of *T*. *gondii*-specific IgG and IgG2a antibody responses compared to naïve challenged group (Naïve Cha) (Fig 2A and 2C, *P < 0.01). Multi-antigen Cha also showed significantly higher levels of *T*. *gondii*-specific IgA antibody responses in feces compared to Combination Cha or Naïve Cha (Fig 2D, *P < 0.01).

### VLPs vaccination induces CD4^+^ T cells and CD8^+^ T cells responses

**Fig 3:**
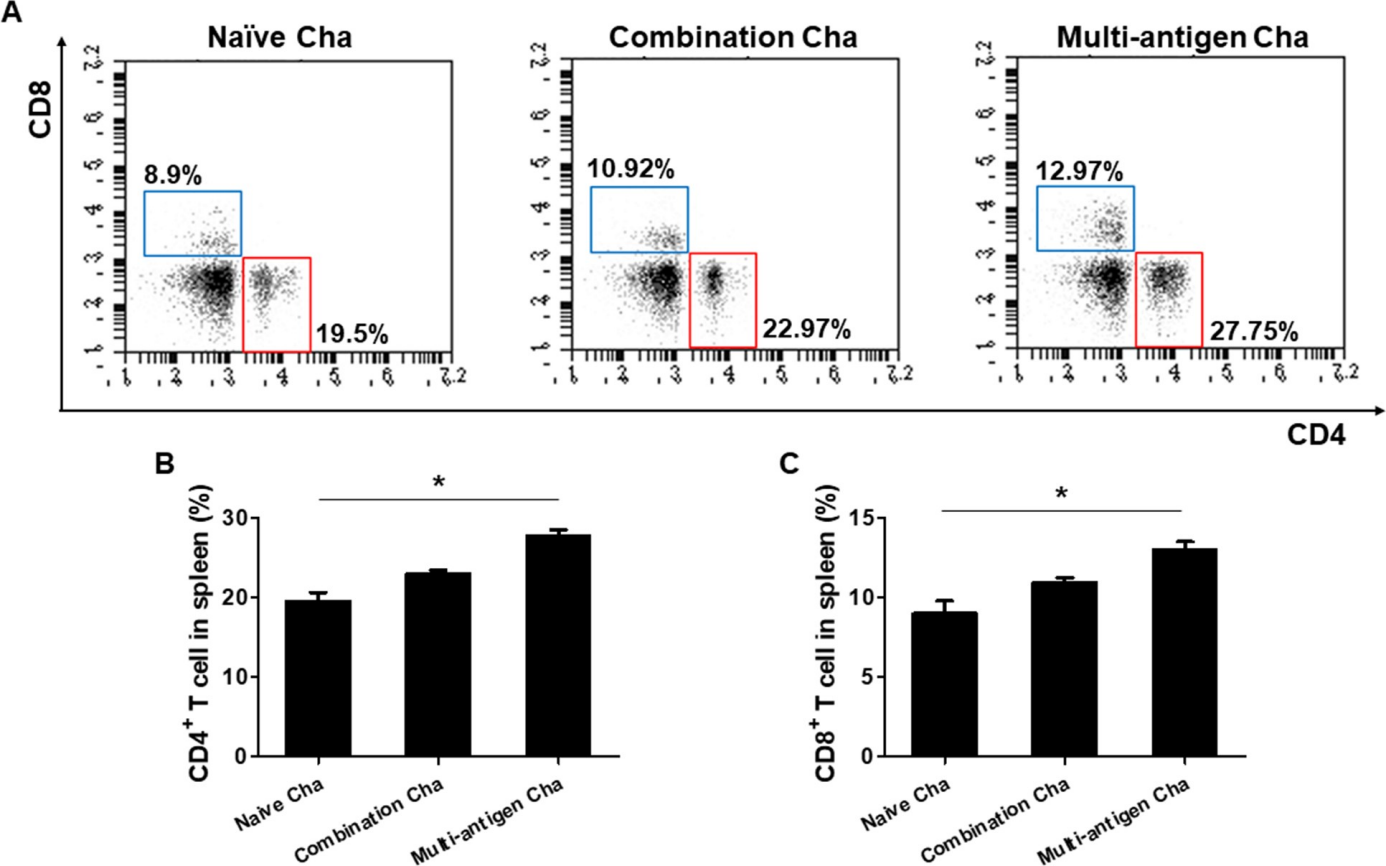
CD4^+^ and CD8^+^ T cell response. To investigate the T cell responses, the splenocytes were prepared from mice spleen and stained with fluorescence conjugated CD3, CD4 and CD8 antibodies (A). The highest levels of CD3^+^, CD4^+^ (B) and CD3^+^, CD8^+^ (C) T cells were found in multi-antigen VLPs immunized mice (Multi-antigen Cha) compared to combination VLPs immunized mice or naïve challenged mice (*P < 0.01).

To determine T cell responses in immunized mice upon challenge, flow cytometer analysis was performed using mouse spleen cells. As shown in Fig 3, at 4 weeks post-challenge, higher populations of CD4^+^ T cells and CD8^+^ T cells were found in multi-antigen VLPs (Multi-antigen Cha, *P < 0.01) or combination VLPs (Combination Cha, *P < 0.01) immunized mice compared to naïve challenge (Naïve Cha) control mice. Significantly higher levels of CD4^+^ and CD8^+^ T cell responses were found in Multi-antigen Cha compared to those in Combination Cha (Fig 3B and 3C, *P < 0.01).

### T and B cell epitopes prediction and VLPs vaccines-induced memory cell responses

As shown in Table 1, many T cell and B cell epitopes from *T*. *gondii* IMC, ROP18 and MIC8 in VLPs were predicted by IEDB tools. The top score (more than 0.7) T cell epitopes (9-mer) were identified and the overlapped B cell epitopes (more than 5 parameters) were selected (Table 1).

**Table 1:** The T cell and B cell epitopes predicted in protein IMC, ROP18 and MIC8.

Epitope		IMC	ROP18	MIC8
T cell epitope		37–45 87–95 101–109	259–267 289–297 344–352 363–371 407–415 463–471	90–98 114–122 231–239 382–390 478–486 559–567 582–590
B cell epitope	Linear epitope	6–18 149–164	7–68	48–85 537–616 623–671
	Beta-turn	4–15 145–146 151–161	43–67	49–58 581–603 628–635
	Exposed surface	146–161	53–65 67–72	46–57 581–603 626–651
	Flexibility	10–15 145–160	51–67	47–58 580–605 628–659
	Antigenicity	4–10 136–145	64–69	-
	Hydrophilicity	7–15 147–161	53–70	49–57 580–602 628–639

T cell and B cell epitope of *T*. *gondii* inner membrane complex (IMC), rhoptry protein 18 (ROP18) and microneme protein 8 (MIC8) were predicted by IEDB online service. T cell epitopes were predicted based on propensity scores of MHC binding affinity, rescale binding affinity, C-terminal cleavage affinity, and transport associated with antigen processing (TAP) transport efficiency. Sequences with the highest total score was selected. B cell epitope was analyzed by assessing the six amino acid biophysical parameters as outlined in the table. Among the numerous sequences with high scores, overlapping sequences over five different methods were selected.

**Fig 4:**
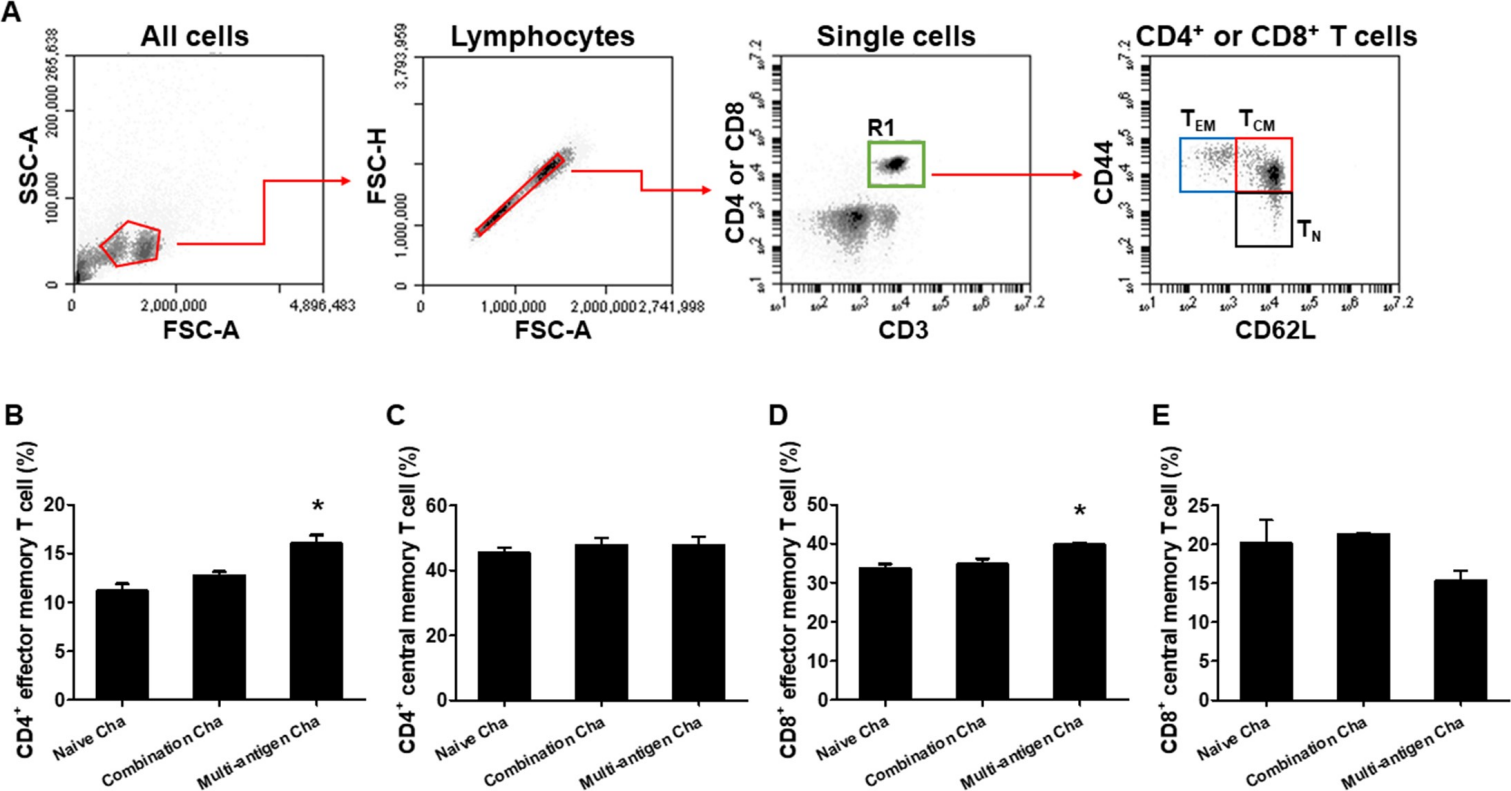
Memory T cell response. Flow cytometry plots showing the gating strategy to identify memory T cell subsets (A). The CD4^+^ and CD8^+^ effector memory T cells were separated by CD3^+^, CD4^+^, CD44^+^ and CD62L^-^ and CD3^+^, CD8^+^, CD44^+^ and CD62L^-^ staining. Significantly higher level of CD4^+^ and CD8^+^ effector memory T cell was found in multi-antigen VLPs immunized mice (Multi-antigen Cha) compared to combination VLPs immunized mice (Combination Cha) or naïve challenged mice (Naïve Cha) (B and D, *P < 0.01). The CD4^+^ and CD8^+^ central memory T cells were separated by CD3^+^, CD4^+^, CD44^+^ and CD62L^+^ and CD3^+^, CD8^+^, CD44^+^ and CD62L^+^ staining. There was no significant difference among the mice groups (C).

**Fig 5:**
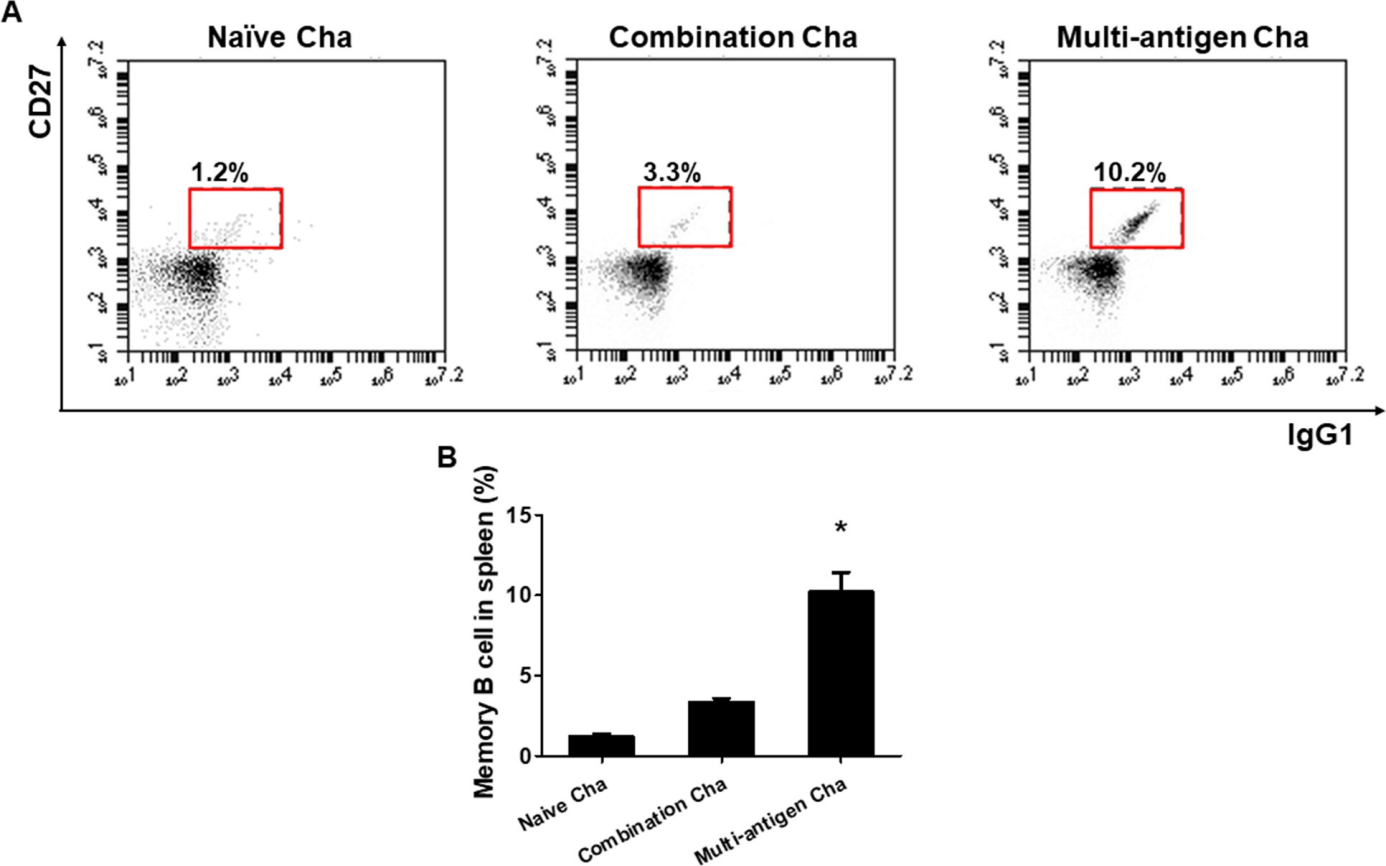
Memory B cell response. The memory B cells responses were determined by flow cytometry analysis. Splenocytes were stained with CD45R/B220, CD27 and IgG1 and gated with CD45R/B220 positive population. The CD45R/B220 positive cells were separated by CD27^+^ and IgG1^+^ staining as memory B cell (A). Higher level of memory B cells responses were found in multi-antigen VLPs immunized mice (Multi-antigen Cha) compared to naïve (Naïve Cha) or combination VLPs immunized mice (Combination Cha) (B, *P < 0.01).

MIC8 of *T*. *gondii* had more T and B cell epitope than IMC and ROP18. VLPs vaccination induced memory T and B cell responses. As shown in Fig 4, Multi-antigen Cha showed significant higher levels of CD4^+^ and CD8^+^ effector memory T cells (T_EM_) responses compared to Combination Cha and Naïve Cha (Fig 4B and 4D, *P < 0.01). However, there was no significant difference in CD4^+^ and CD8^+^ central memory T cell (T_CM_) responses between the groups (Fig 4C–4E). Significantly higher level of memory B cells in Multi-antigen Cha compared to Combination Cha and Naïve Cha were found (Fig 5, *P < 0.01).

### VLPs vaccination induces antibody secreting cell responses

**Fig 6:**
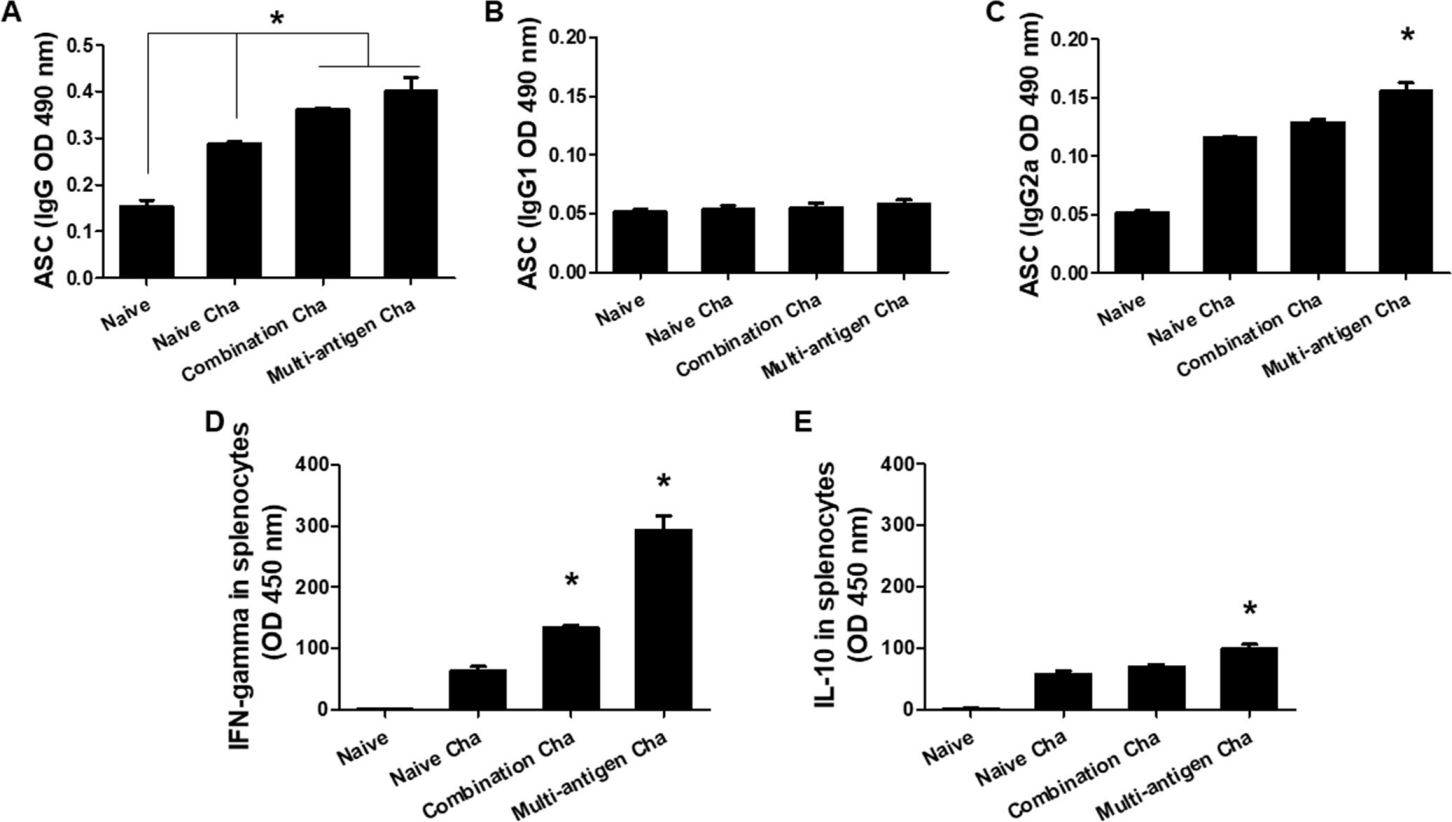
Antibody secreting cells (ASC) and cytokines responses. Spleen cells (10^6^ cell/well) from mice were cultured and the level of IgG, IgG1 and IgG2a antibody secreting cells were determined (A, B and C). Higher level of *T*. *gondii*-specific IgG was found in multi-antigen or combination VLPs immunized mice (Multi-antigen Cha or Combination Cha) compared to Naïve mice (Naïve Cha) (A, *P < 0.01). The highest level IgG2a was found in Multi-antigen Cha mice compared to the other mice groups (C, *P < 0.01). To detect cytokines (IFN-γ and IL-10), splenocytes were cultured with *T*. *gondii* antigen (5 μg/mL) (D and E). Significantly higher levels of IFN-γ and IL-10 were detected in Multi-antigen Cha compared to the other mice groups (D and E, *P < 0.01).

As shown in Fig 6, higher level of IgG antibody secreting cell responses was found in multi-antigen VLPs (Multi-antigen Cha) or combination VLPs (Combination Cha) immunized mice compared to that of naïve challenged group (Naïve Cha, Fig 6A, *P < 0.01). Significantly higher level of IgG2a antibody response was detected in Multi-antigen Cha compared to the other mice groups (Fig 6C, *P < 0.01).

### VLPs vaccination induces cytokine responses

The splenocytes from VLPs immunized mice were cultured to determine the production of Th1 and Th2—like cytokines. Multi-antigen VLPs (Multi-antigen Cha) vaccination induced significantly higher levels of IFN-γ and IL-10 compared to combination VLPs (Combination Cha) or naïve challenged control mice (Naïve Cha) (Fig 5D and 5E, *P < 0.01). Higher level of IFN- γ was detected than that of IL-10 (Fig 6D and 6E).

### VLPs vaccinations significantly inhibit *T*. *gondii* replication and protect mice from *T*. *gondii* challenge infection

**Fig 7:**
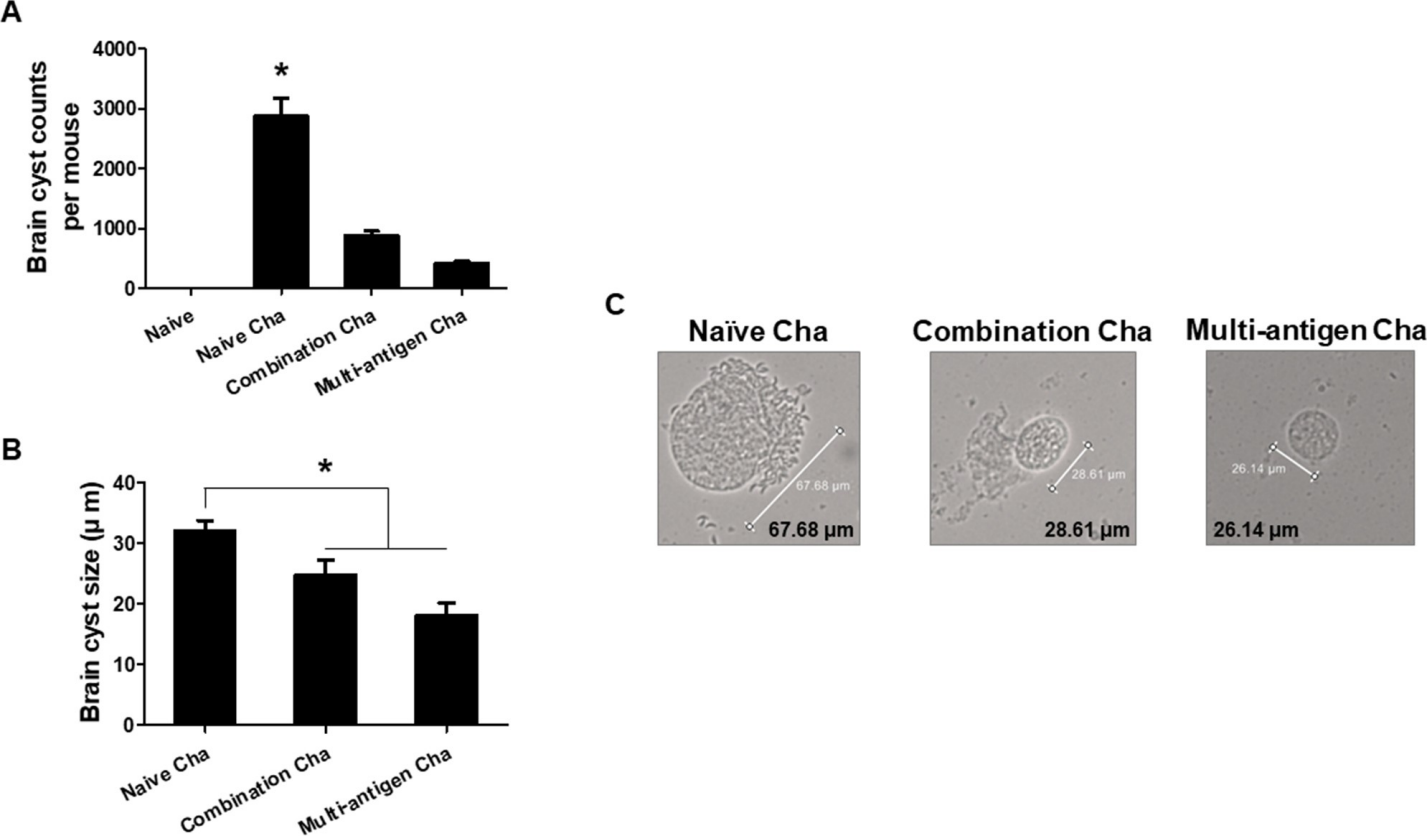
Parasite inhibition. To investigate the vaccine efficacy, mice brain was collected at week 4 after challenge infection. *T*. *gondii* (ME49) were separated individually from mice brain and counted under the microscope. The number of brain cysts was significantly reduced in multi-antigen or combination VLPs immunized mice compared to naïve mice upon challenge infection (A, *P < 0.01). The cysts size was significantly reduced in Multi-antigen Cha (18 μm) or combination Cha (24.8 μm) compared to Naïve Cha (32 μm) (B and C, *P < 0.01).

**Fig 8:**
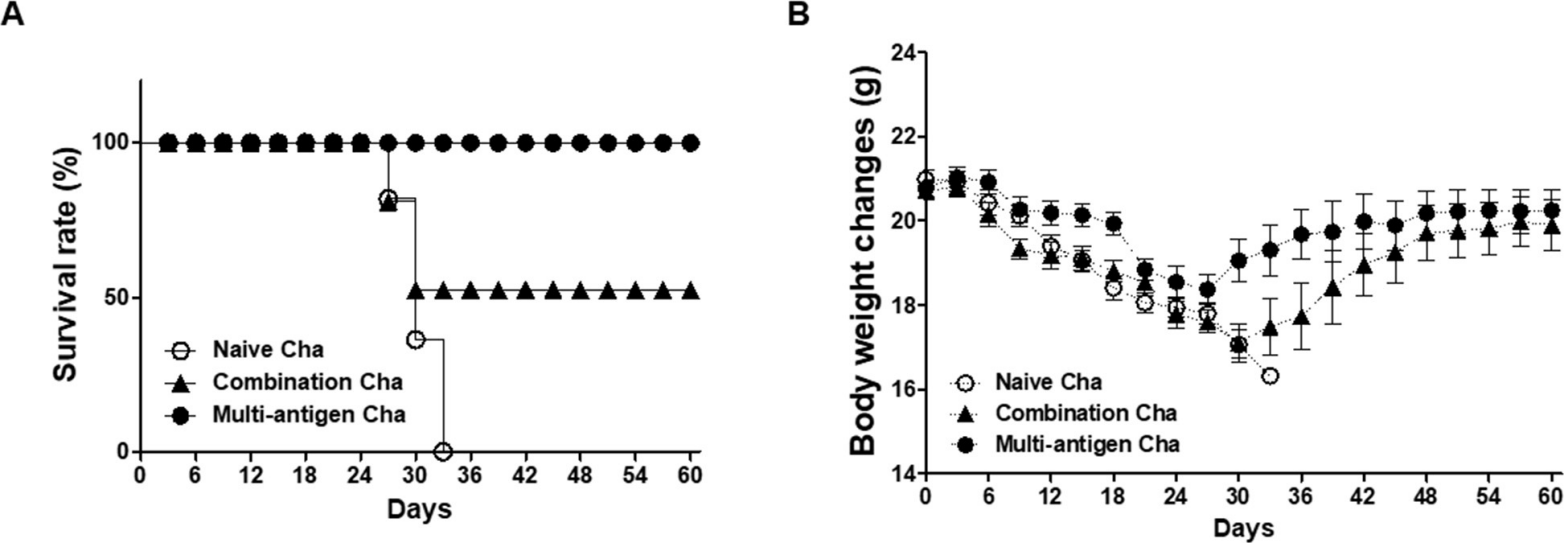
Protection induced by multi-antigen VLPs. All mice survived in multi-antigen immunized mice (Multi-antigen Cha, 100% of survival) whereas naïve mice showed 0% of survival and combination VLPs showed 50% of survival (A). Mice immunized with multi-antigen VLPs showed significantly lower body weight loss compared to naïve mice or combination VLPs (B).

Mice immunized with VLPs were challenge infected with *T*. *gondii* (ME49) and the cysts of *T*. *gondii* in the brain were observed. As shown in Fig 7A, the lower number of cysts was found in multi-antigen VLPs immunized mice (Multi-antigen Cha) compared to combination VLPs immunized mice (Combination Cha) or naïve challenged control mice (Naïve Cha) (Fig 7A, *P < 0.01). Cysts size was found to be significantly reduced in Multi-antigen Cha and Combination Cha compared to Naïve Cha (Fig 7B and 7C, *P < 0.01). The mice were monitored daily to observe the survival rates and body weight changes upon challenge infection. As shown in Fig 8A and 8B, all mice immunized with multi-antigen VLPs showed 100% of survival whereas combination VLPs showed 50% and naïve mice showed 0% of survival. Multi-antigen VLPs showed lower levels of body weight loss compared to combination VLPs at day 27 and 30 post-challenge infection.

## Discussion

Vaccination is the most effective method in protecting the host against infectious diseases [30]. Although the efforts to develop vaccine against *T*. *gondii* infection were made for decades, efficacy issues have hindered its development [22,31]. *T*. *gondii* vaccine-induced memory T and B cell responses and related humoral and cellular immunity study are largely lacking. In the present study, multiple antigenic proteins containing influenza VLPs vaccine were found to be completely protective and correlated with memory T and B cell responses. The VLPs we generated targeted *T*. *gondii* multiple antigenic proteins, which possess T and B cell epitopes.

A highly effective vaccine must be capable of eliciting strong antigen-specific antibody responses as well as ensuring development of persisting memory B and T cells [30,32]. Antibody production post-vaccination is of utmost importance, as their contribution to immunity and protection against pathogens are crucial for controlling disease progression [33]. In our current study, we found that multi-antigen VLPs elicited significantly higher levels of *T*. *gondii*—specific total IgG and IgG2a isotype antibody secreting cells cultured *in vitro* compared to combination VLPs, correlating with higher levels of IgG and IgG2a antibody responses in sera. The dominant IgG2a isotype antibody response is known to be more effective than other isotypes in pathogen clearance [34–36], and their role in complement activation, antibody-dependent cellular cytotoxicity induction, as well as virus removal via macrophages have already been documented [37].

Although memory B cells may not confer direct protection against pathogenic diseases, they appear to be invoked upon depletion of pre-existing antibody levels which are required to prevent infection [30]. Antibody responses detected in sera upon VLPs vaccination were low [25] and increased upon *T*. *gondii* ME49 challenge infection in our current study. Memory B cells were found to be increased upon challenge infection, indicating memory B cell response was initiated because of low level of antibody response which were not able to provide protection against *T*. *gondii* infection as a critical first line of defense.

Protection against intracellular pathogens requires T cell-mediated responses [38]. T cell differentiation and memory and effector T cells play a significant role in immunity against pathogenic agents [39]. Upon exposure to pathogen, memory T cells from vaccination undergo rapid clonal expansion and induce effective immune response compared to response from the primary immune response [39]. Memory T cell populations have been further divided into “central memory” (T_CM_) or “effector memory” (T_EM_) subsets that differ by phenotype and function [40,41]. The importance of T_CM_ or T_EM_ subsets with regards to vaccine-mediated protection against viral challenge infection remains elusive [30]. In our current study, we found that multi-antigen VLPs showed higher levels of both CD4^+^ and CD8^+^ effector memory T cells compared to central memory T cells, indicating that effector memory T cell responses play an important role in contributing the protection against *T*. *gondii* infection. Combination VLPs showed no significant increase in CD4^+^ and CD8^+^ effector memory T cells and central memory T cell responses which might contribute to the low levels of protection induced by combination VLPs. These results may be attributed to higher density or purity of *T*. *gondii* virus-like particles, which were observed in multi-antigen VLPs compared to combination VLPs [25].

Vaccines containing T or B cell epitopes can elicit both cellular and humoral immune responses, the T and B cell epitopes prediction is important in designing epitope-based vaccines [42–45]. In the current study, we found a number of T or B cell epitope regions from antigenic proteins of IMC, ROP18 and MIC8 from *T*. *gondii* by epitope prediction tools. Epitope prediction results outlined in Table 1 have revealed that MIC8 contained more T and B cell epitope regions than other antigens, indicating its expression in the VLPs might have contributed more to immunological protection than others. Since MIC8 VLPs vaccination has shown great protection against highly virulent *T*. *gondii* RH challenge infection [22], T and B cell epitope portions from MIC8 could be used for epitope—based vaccine designs.

## Conclusions

Multi-antigen VLPs vaccination induced significantly higher levels of CD4^+^ CD8^+^ effector memory T cell and memory B cells compared to combination VLPs. T and B cell epitopes from IMC, ROP18 and MIC8 in multi-antigen VLPs might contribute to induce memory T and B cells responses, which result in effective CD4^+^, CD8^+^ T cell responses, antibody secreting cell responses, IgG, IgG2a, IgA antibody responses in sera or feces and complete protection against *T*. *gondii* ME49 challenge infection.

## Supporting information

S1 Table (DOCX)

S2 Table (DOCX)

S3 Table (DOCX)
