# Supplementary material for: Virus-like particles containing multiple antigenic proteins of Toxoplasma gondii induce memory T cell and B cell responses
Source: PLoS One. 2019 Aug 29;14(8):e0220865. doi: 10.1371/journal.pone.0220865 (PMC6715270; doi:10.1371/journal.pone.0220865)
Supplement: S1 Table — (DOCX) [file pone.0220865.s001.docx]

**S1 Table. The T and B cell epitopes predicted in inner membrane complex**

| Epitope | | Sequence | Position |
| --- | --- | --- | --- |
| T cell epitope | | **AFDNWMNRY**  **LDDIAAVLY**  **SSECADSKM** | **37-45**  **87-95**  **101-109** |
| B cell epitope | **Linear epitope 1** | **FDSDSTADLEIGREGEVRSRKPIQV**  **KE, VEAGD**  **R, D, GSDPRSSECA**  **IKKNQESNKSSASNDRN** | **9-33**  **35-36, 45-49**  **60, 68, 96-105**  **149-164** |
|  | **Linear epitope 2** | **CCGFDSDSTADLE**  **KPIQVSKEAFDNWMNRYEAGDT**  **NFMNLTFNQKVRPI, AA**  **LVGSDPRSSECADSKMLRNP**  **RLASSGRAIAFSFK**  **EIKKNQESNKSSASND** | **6-18**  **29-50**  **72-85, 91-92**  **94-113**  **119-132**  **147-162** |
|  | **Beta-turn** | **TACCGFDSDSTA**  **R, K, A**  **NWMN, YEAG**  **PDGHRI, KI**  **RPKNF, F, Q**  **YGSDPRSSECA**  **MLR, CV**  **LASSGR**  **D, D, IK**  **NQESNKSSASN** | **4-15**  **26, 29, 37**  **40-43, 45-48**  **56-61, 66-67**  **69-73, 78, 83**  **95-105**  **109-11, 114-5**  **120-125**  **133, 136, 145-6**  **151-161** |
|  | **Exposed surface** | **GEVRSRKP, WMNRVEA**  **KIDRPKN, FNQKVR**  **DEIKKNQESNKSSASN** | **23-30, 41-47**  **66-72, 78-83**  **146-161** |
|  | **Flexibility** | **T, DSDSTA**  **GREGEVRSRKPI**  **SKE, AGD**  **KIDRPK, NQKVRP**  **GSDPRSSECADSKMLRN**  **SSGR, KDITD**  **DDEIKKNQESNKSSAS** | **4, 10-15**  **20-31**  **34-36, 44-46**  **66-71, 79-84**  **96-112**  **122-5, 132-6**  **145-160** |
|  | **Antigenicity** | **TACCGFD, PIQVSKE**  **EVLFPD**  **KVRPIQLDDIAAVLVGS**  **RNPCVVGFRLA**  **AIAFSF**  **DAQCFVSFLD** | **4-10, 30-36**  **52-57**  **81-97**  **111-121**  **126-131**  **136-154** |
|  | **Hydrophilicity** | **TA, CGFDSDSTA**  **I, REGEVRS, K**  **VS, EA**  **YEAGDT, HRI**  **R, SDPRSSECAD**  **M, SSGR**  **D, TD**  **EIKKNQESNKSSASN** | **4-5, 7-15**  **19, 21-27, 29**  **33-34, 36-37**  **45-50, 59-61**  **69, 97-106**  **109, 122-125**  **133, 135-136**  **147-161** |

T cell and B cell epitopes of *T. gondii* inner membrane complex (IMC) were predicted by IEDB online service. T cell epitope was predicted score of MHC binding affinity, rescale binding affinity, c terminal cleavage affinity and tap transport efficiency. B cell epitope was analyzed by 7 methods, linear epitope 1, linear epitope 2, bera-turn, exposed surface, flexibility, antigenicity and hydrophilicity.
