# Supplementary material for: Virus-like particles containing multiple antigenic proteins of Toxoplasma gondii induce memory T cell and B cell responses
Source: PLoS One. 2019 Aug 29;14(8):e0220865. doi: 10.1371/journal.pone.0220865 (PMC6715270; doi:10.1371/journal.pone.0220865)
Supplement: S2 Table — (DOCX) [file pone.0220865.s002.docx]

**S2 Table. T and B cell epitopes predicted in rhoptry protein 18**

| Epitope | | Sequence | Position |
| --- | --- | --- | --- |
| T cell epitope | | **GSGGFATVY**  **ATDVETNEE**  **PTDETMLDL**  **LTTRWVPNY**  **MSKVISWVF**  **HTDIKPANF**  **PTDAWQLGI** | **259-267**  **269-277**  **289-297**  **344-352**  **363-371**  **407-415**  **463-471** |
| B cell epitope | **Linear epitope1** | **RPPLT**  **GTGITLGPSKLDSKPTSLDSQQHV**  **GATESTR**  **ERDGEVSGSAADSSSRPR**  **PQGRNRQ, QIGQPQALENS**  **AWPPDVPKR**  **TGETRT**  **PLGSGGFA**  **EATDVETN**  **E, EKEPTDET**  **S, TAKDAQE**  **GQPAST**  **ASVNK, I**  **GRAIGTPGVEPPERPFQA**  **VTFPTD, ERPTPADG**  **CPSTPEL, RDPQK**  **KG**  **AQNFEQ, HL** | **6-10**  **45-68**  **85-91**  **101-111**  **171-7, 211-21**  **232-240**  **246-251**  **257-264**  **268-275**  **277, 286-293**  **302, 312-318**  **333-338**  **374-378, 410**  **438-455**  **460-5, 480-7**  **497-503, 514-8**  **539-540**  **542-7, 550-1** |
|  | **Linear epitope2** | **PPLTRTVVRMGLATLLPKTACLAGLNVALVFLL**  **FQVQDGTGITLGPSKLDSKPTSLDSQQHVADK**  **VGHVKHLAGATESTRDVSLLEE**  **RAQHRVNAQETNQ**  **RDGEVSGSAADSSSRP**  **LRAQRRRSELVFEKADSGCVI**  **RILAHMQEQIGQPQALENSERL**  **KRFVSVTTGETRTLVRGAPLGSGGFATVVEATDVETNEEL**  **AVKVFMSEKEPTDETMLDLQRESSCVRNFSLAKTAKDAQ**  **ESCRFMVPSDVVMLEGQPASTEVVIGLTT**  **V**  **RAEADMSKVISWVFGDASVNKSEFGLVVRMVL**  **SSQAIKLVANVQAQGIVHTDIKP**  **TVRINNSVGRAIGT**  **I, VTFPTDAWQL**  **ITLVCIWCKERPT**  **D, PELV**  **D, QKRMLPLQA**  **ETAAFKEMDSVVKGAAQNFEQQEHL** | **7-71**  **77-111**  **128-143**  **180-200**  **203-224**  **239-346**  **349**  **358-412**  **430-443**  **458, 460-469**  **471-483**  **490, 501-504**  **506, 517-525**  **527-551** |
|  | **Beta-turn** | **QRPPLT, A**  **QDGTGITLGPSKLDSKPTSLDSQQH**  **GH, KH**  **TESTRD, QETNQR**  **ERDGEVSGSAADSSSRPRLS**  **F, I, R**  **FPQGRNRQR**  **ADSGCVK**  **IGQPQAL, ER**  **AWPPDVP, T**  **ET, R**  **APLGSGGFA**  **VE**  **KEPTDET**  **QRESSCYRNFSL**  **K**  **AQE, FMV**  **QPAS, WVPN**  **K, VFGDASVNKSE**  **LS, QA**  **V, DIKP**  **KDGRLF**  **GDFGTYRINNSVGR**  **IGTPGYEPPERP**  **QA, GI**  **Y, FPTDAW**  **KERPTPADGIWD**  **H, ADCPSTPEL**  **LNRDPQKR**  **VV, AAQ** | **5-10, 29**  **43-67**  **78-9, 81-2**  **87-92, 107-12**  **127-146**  **162, 166, 168**  **170-178**  **194-200**  **212-8, 222-3**  **232-238, 246**  **248-249, 254**  **256-264**  **269-270**  **287-293**  **298-309**  **314**  **316-8, 322-4**  **334-7, 348-51**  **365, 370-380**  **388-90, 401-2**  **406, 409-412**  **419-424**  **426-439**  **441-452**  **454-5, 457-8**  **460, 462-467**  **479-490**  **493, 495-503**  **512-519**  **537-8, 541-3** |
|  | **Exposed surface** | **SKLDSKPTSLDSQ**  **HVADKR**  **ERAQHRVNAQETNQRRTI**  **LRRRERDG**  **RVFPQGRNRQRSLRAQRRRS**  **QALENSERL**  **SEKEPTD**  **LQRESS**  **PGVEPPERPF**  **KERPTP**  **NRDRQKR** | **53-65**  **67-72**  **98-115**  **123-130**  **168-187**  **216-224**  **285-291**  **297-302**  **444-453**  **479-484**  **513-519** |
|  | **Flexibility** | **QRPPLTR, PKT**  **QDGTGI**  **GPSKLDSKPTSLDSQQH**  **DKR, ATESTRD**  **EER, AQETNQRRT**  **RERDGEVSGSAADSSSRPR**  **VRQR**  **RAKSLFKRGIR, FPQGRNRQRSL**  **AQRRRSE**  **EKADSG**  **EQIGQPQALENSERLD**  **PPDVPKR**  **TTGETRT**  **APLGSGG**  **ATDVETNEE, SEKEPTDET**  **QRESS**  **KTAKDAQES, PSD**  **EGSPAST, TR**  **DASVNKSE, SSQ**  **DIKPA**  **KDGR**  **DFG, MNSVG**  **GTPGYEPPERP**  **TG, PTD**  **ERPTPADG**  **CPSTPEL, NRDPQKR**  **EMDS, FEQQEH** | **5-11, 23-25**  **43-48**  **51-67**  **70-72, 86-92**  **97-9, 106-14**  **126-144**  **144-147**  **157-67, 170-80**  **182-188**  **192-197**  **210-225**  **234-240**  **245-251**  **256-262**  **269-77, 285-93**  **298-302**  **311-9, 325-7**  **332-8, 346-7**  **373-380, 390-2**  **409-413**  **419-422**  **427-9, 434-8**  **442-452**  **456-7, 463-5**  **480-487**  **497-503, 513-9**  **530-3, 545-50** |
|  | **Antigenicity** | **ATLLPKTACLAGLNVALVFLLFQV**  **SQQHVA**  **LATVGHVKHL**  **QRLLNL**  **RLSVRQRLAQL**  **SGCVIGKRILAH**  **ILTVAAWPPDVPKRFVSVT**  **PSDVVMLE**  **STEVVIGL**  **FGLVVRMY**  **SSQAIKLVANV**  **AQGIVHTD**  **ANFLLLKD**  **GITLVCIWC**  **DVLHFADCP**  **TPELVQDLIRS**  **MLPLQALET**  **DSVVKG** | **19-42**  **64-69**  **74-83**  **117-122**  **144-154**  **196-207**  **227-245**  **325-332**  **337-344**  **381-388**  **390-400**  **402-409**  **413-420**  **470-478**  **490-498**  **500-510**  **520-528**  **535-540** |
|  | **Hydrophilicity** | **PL, C**  **QDGTGI**  **SKLDSKPTSLDSQQHVAD**  **GH, AGATESTRD**  **L, E**  **RAQHRVNAQETNQR**  **RERDGEVSGSAADSSSRPR**  **SV, Q, I**  **FPQGRNRQRSLRAQRRRS**  **KADSGCVI**  **MQEQIGQ**  **QALENSERL, VP**  **TTGETRTL**  **PLG, GF**  **YEATDVETNEEL**  **SEKEPTDETM**  **QRESSCYR**  **KTAKDAQESC**  **GQPAST, W**  **AEADM, K**  **ASVNKSE**  **VQA, G, V**  **DIK, G**  **RINNSVGRAIGTPGYEPPERPFQ**  **GI, FP**  **ERPTPA, G**  **DCPSTPEL**  **LNRDPQK**  **E, A**  **KEMDSVVKGAAQNFEQQEHL** | **8-9, 27**  **43-48**  **53-70**  **78-79, 84-92**  **95-97**  **99-112**  **126-144**  **146-7, 149, 166**  **170-187**  **193-200**  **208-214**  **216-24, 237-8**  **245-252**  **257-9, 262-3**  **267-278**  **285-294**  **298-305**  **311-320**  **333-338, 348**  **359-363, 365**  **374-380**  **400-2, 404, 406**  **409-411, 429**  **432-454**  **457-8, 462-3**  **480-485, 487**  **496-503**  **512-518**  **527-530**  **532-551** |

T cell and B cell epitopes of *T. gondii* rhoptry protein 18 (ROP18) were predicted by IEDB online service. T cell epitope was predicted score of MHC binding affinity, rescale binding affinity, c terminal cleavage affinity and tap transport efficiency. B cell epitope was analyzed by 7 methods, linear epitope 1, linear epitope 2, bera-turn, exposed surface, flexibility, antigenicity and hydrophilicity.
