# Supplementary material for: Virus-like particles containing multiple antigenic proteins of Toxoplasma gondii induce memory T cell and B cell responses
Source: PLoS One. 2019 Aug 29;14(8):e0220865. doi: 10.1371/journal.pone.0220865 (PMC6715270; doi:10.1371/journal.pone.0220865)
Supplement: S3 Table — (DOCX) [file pone.0220865.s003.docx]

**S3 Table. T and B cell epitopes predicted in microneme protein 8**

| Epitope | | Sequence | Position |
| --- | --- | --- | --- |
| T cell epitope | | **CCSKTACFY**  **SSIIYHDEY**  **TCTRTDEGY**  **NTDDSYECV**  **QCYSHGTSY**  **YTEVFVNGK**  **ECTTDGTAY**  **YSDYVKGCV**  **SSDAVDACI**  **YTCRCKDGY**  **STDQTDFDS** | **90-98**  **114-122**  **181-189**  **231-239**  **366-374**  **382-390**  **412-420**  **478-486**  **545-553**  **559-567**  **582-590** |
| B cell epitope | **Linear epitope1** | **Q, KEPPDPDDENS**  **DACGSREVSDKGLKGTVC**  **W, YSY**  **NREDGGAVC**  **GVWGDGKS**  **CGAGTCTRTDEGVKCDCP**  **KETCKAKPDFCAEEPCGPPSMV**  **VNTDDSY, VEVRNG**  **C, DKPCGPDEGVH**  **TERQPK, TLPDGVS**  **EPCGTR, SKA**  **RNDPCGPETA**  **SSCQKGDPC**  **GNEAVQECTTDGTAVGCTCK**  **G, KFCNPEEECASH**  **GVQCTC, VEG**  **QCSLNPCGEQEA**  **CIPEGDTVDCEC**  **I, ASCVGNPCGSSDAV**  **KDGVTPQSIG**  **PESTDQTDFDSKHKPEDNKGRVSK**  **SVARNRGGERDDEDLAPPPRSTRER**  **EGFENASWA**  **MIPSAPAPPPSGGIW** | **37, 48-58**  **68-85**  **104, 122-4**  **146-154**  **159-166**  **177-194**  **205-226**  **230-6, 244-9**  **251, 259-69**  **273-8, 293-9**  **310-5, 323-5**  **354-363**  **391-399**  **406-425**  **427, 437-48**  **465-70, 486-8**  **490-501**  **505-516**  **531, 539-49**  **564-573**  **580-603**  **625-649**  **656-664**  **669-683** |
|  | **Linear epitope2** | **LKEMDSIFVSAIRQNVQHTHSALL**  **DENSWLCRISKKVDACGSREVSDKGLK**  **DFCCSKTACFVGSCFSWCHDNWALCSSSIIVHD**  **YGKCNKRFQENCDVNAICVHANR**  **EDGGAVCQCKEGVWGDGKSCKID**  **GAGTCTRTDEGVKCDPETHKLIVVEDKETCKAK**  **SMVENCVNTDDSVECVCKQGVEVRNGRCEEIDL**  **D**  **GPDEGVHECVT**  **RCTCKAGFDLTTLPDGVSQKCLKN**  **V**  **LVESCKSKAVGVSCLCAAGAMVQVINGKEKCIK**  **RN**  **TAVIQCVSHGTSVRCLCKAGVTEVFVNGKSSC**  **QKGDPCTLNMCGGNEAVQECTTDGTAVGCTCK**  **PGVSIAIKHGQKFCNPEEECASHCGSAAAVK**  **SCEILDSGGVQCTCNPGVVMRVSDVVKGC**  **VEGNQCSLNPCGEQEAVQR**  **PEGDTVDCEC**  **A**  **SCVGNPCGSSDAVDACIAGTSTVTCRCKDG**  **YTPQSIGSKLQCLPESTDQTDFSKHKP**  **EDNKGRVSKGTIALVVVGCVAL**  **G**  **GISVARNRGGERDD**  **STRERRLSSMGEGFENASWASSVSMIP**  **PPSG** | **21-44**  **55-81**  **88-120**  **124-170**  **178-211**  **224-256**  **259**  **263-273**  **282-305**  **308**  **317-349**  **354-355**  **362-504**  **507-516**  **533**  **537-616**  **618**  **623-636**  **645-671**  **677-680** |
|  | **Beta-turn** | **EPPDPDDENS**  **YDACGSREYSDKGLKGTYCPEDFCC**  **FYGSCGSWCHD**  **ALC, EYSYGKCNK**  **E, CD**  **REDGGAYC**  **CKEGYWGDGKSC**  **QPCGAGT, T**  **TDEGYCDCPE, A**  **EPCGPPSMV**  **VNTDDSYE**  **K, V**  **R, ADKPCGPDE**  **C, TLPDGVS**  **C, Y**  **EPCGT, SKAYGYSC**  **KE, CRNDPCGP**  **YSHGTSY, C**  **GKSSCQKGDPCTLNMCGGNE**  **TTDGTAYGCTCKPGY**  **KFC, E**  **SHCG**  **DSGGYQCTCNPGY, DYV**  **VEGNQCSLNPCG**  **PEGDTYDCECNPGF**  **LPDGNFI, P**  **SCVGNPCGSSDA**  **STYT**  **RCKDGYTPQSI**  **ESTDQTDFDSKHKPEDNKGRYSK**  **ISY, RNRGGERD**  **DLAPPPRST** | **49-58**  **67-91**  **97-107**  **110-2, 121-30**  **134, 136-137**  **147-154**  **156-167**  **178-181, 183**  **185-195, 210**  **218-226**  **230-237**  **241, 246**  **250, 258-266**  **285, 293-299**  **302, 308**  **310-4, 323-30**  **344-5, 353-60**  **368-374, 376**  **389-408**  **414-428**  **437-439, 442**  **447-450**  **462-74, 480-2**  **486-497**  **507-520**  **525-531, 535**  **537-548**  **557-560**  **562-572**  **581-603**  **624-6, 628-35**  **638-646** |
|  | **Exposed surface** | **QNVQHT**  **KLKEPPDPDDEN**  **REVSDKGL**  **ANREDG, TRTDEGV**  **EDKETCKAK**  **QGVEVR, ERQPKLR**  **MRVSDV**  **ESTDQTDFDSKHKPEDNKGRVSK**  **VARNRGGERDDEDLAPPPRSTRERRL**  **APAPPP** | **34-39**  **46-57**  **73-80**  **145-50, 183-9**  **203-211**  **242-7, 274-80**  **476-481**  **581-603**  **626-651**  **673-678** |
|  | **Flexibility** | **KEMD, RQN**  **LKEPPDPDDEN, SKK**  **GSREYSDKGLKGTYCPED**  **KT, SCG**  **SS, K**  **KRFQEN, REDGG**  **KEG, GDGKSCK**  **PCGAGTCTRTDEGYK**  **CPETH, EDKETCKAKPD**  **EEPCGPP**  **TDDS, KQGY**  **VRNGRCEE**  **DKPCGPDEG, TERQPK**  **TTLPDGVSQKC**  **EEPCGTRDL**  **ESCKSKA**  **NGKEK, RNDPCGPET**  **GTS, NGKSSCQKGDP**  **GGNEA, ECTTDGT**  **KPG, HGQK**  **NPEEE, LDSGG**  **DY, VEGNQC**  **PCGEQEA**  **PEGDT, LPDGN**  **DPA, GNPCGSSDA**  **GTSTY, KDGYTPQSIGSK**  **PESTDQTDFDSKHKPEDNKGRYSKGT**  **RNRGGERDDEDLAPPPRSTRERRLSSMGEGFE**  **SAPAPPPSG** | **22-25, 33-35**  **47-58, 64-66**  **71-88**  **93-4, 100-2**  **113-4, 126**  **130-5, 147-51**  **157-9, 162-8**  **176-190**  **190-4, 203-13**  **217-223**  **232-5, 241-4**  **246-253**  **259-67, 273-8**  **292-302**  **309-317**  **319-325**  **342-6, 354-62**  **371-3, 388-98**  **405-9, 412-8**  **425-7, 434-7**  **440-4, 461-5**  **480-1, 486-91**  **495-501**  **507-11, 525-9**  **534-6, 540-8**  **555-9, 564-75**  **580-605**  **628-659**  **672-680** |
|  | **Antigenicity** | **QHTHSALLAKL**  **PEDFCCSKTACFVGS**  **LCSSSII, VNAICVHA**  **KIDFCQLQPC**  **KLIVVE**  **VECVCKQG**  **IDLCADK, RVRCTCK**  **SQKCLKNF**  **GVSCLCAAG**  **AVIQCVSH**  **SVRCLCKA, GCTCKPG**  **ASHCGSAAAVKSCEIL**  **QCTCNPG, DVVKGC**  **CADPASCVGNP**  **VDACIAG**  **KLQCLPE**  **IALVVVGCVALLGI** | **37-47**  **86-100**  **111-7, 138-45**  **168-177**  **198-203**  **236-243**  **254-60, 280-6**  **299-306**  **327-335**  **363-370**  **373-80, 421-7**  **446-461**  **467-73, 480-5**  **532-542**  **549-555**  **575-581**  **606-619** |
|  | **Hydrophilicity** | **VQHT, EPPDPDDEN**  **KKYDACGSREYSDKG**  **YC, FCC**  **K, C**  **EYSYGKCN, RFQENCD**  **ANREDGGAYCQCK**  **YWGDGKSC**  **GAGTCTRTDEGYKCDCPE**  **DKETCKA, FCA**  **EPCG**  **CVNTDDSYE, C**  **QGYEVRNGRCE, A**  **KPCGPDEGV, VTERQP**  **CTC, A**  **VS**  **C, C**  **EPCGT, DL**  **E, CKSKAY**  **NGKEKCI, DPCGPE**  **SHGTS, GY**  **GKSSCQKGDPC**  **GGNEAVQECTTDGTAYGCTCKP**  **H, KFCNPEEECASHCGS**  **AA, D**  **GGYQCTCN, YV**  **CVEGNQCS**  **PCGEQEAV**  **IPEGDTYDCEC**  **PASCVGNPCGSSDAV**  **I, G**  **STYTCRCKDGYT, SI**  **PESTDQTDFDSKHKPEDNKGRYS**  **RNRGGERDDEDL**  **PRSTRERR, MGEGFE**  **SW, PAPPP** | **36-39, 49-57**  **65-80**  **84-85, 89-91**  **93, 105**  **121-8, 131-7**  **145-157**  **160-167**  **178-195**  **204-10, 214-6**  **218-221**  **229-237, 240**  **242-252, 258**  **260-8, 272-7**  **283-285, 287**  **298-299**  **302, 307**  **310-4, 316-7**  **319, 321-326**  **342-8, 356-61**  **369-73, 381-2**  **389-399**  **405-426**  **434, 437-451**  **453-454, 462**  **464-71, 481-2**  **485-492**  **495-502**  **506-516**  **535-549**  **553, 555**  **557-68, 571-2**  **580-602**  **628-639**  **643-50, 654-9**  **662-3, 674-8** |

T cell and B cell epitopes of *T. gondii* microneme protein 8 (MIC8) were predicted by IEDB online service. T cell epitope was predicted score of MHC binding affinity, rescale binding affinity, c terminal cleavage affinity and tap transport efficiency. B cell epitope was analyzed by 7 methods, linear epitope 1, linear epitope 2, bera-turn, exposed surface, flexibility, antigenicity and hydrophilicity.
